# Decision making on helminths in cattle: diagnostics, economics and human behaviour

**DOI:** 10.1186/s13620-016-0073-6

**Published:** 2016-09-27

**Authors:** Johannes Charlier, Valérie De Waele, Els Ducheyne, Mariska van der Voort, Fiona Vande Velde, Edwin Claerebout

**Affiliations:** 1 Avia-GIS, Risschotlei 33, 2980 Zoersel, Belgium; 2 Chair Group Business Economics, Wageningen University, Hollandseweg 1, 6706 KN Wageningen, The Netherlands; 3 Department of Virology, Parasitology and Immunology, Faculty of Veterinary Medicine, Ghent University, Salisburylaan 133, 9820 Merelbeke, Belgium

**Keywords:** Cattle, Helminth, Diagnosis, Nematode, Liver fluke, Helminth, Economics, Communication

## Abstract

Helminth infections of cattle affect productivity in all classes of stock, and are amongst the most important production-limiting diseases of grazing ruminants. Over the last decade, there has been a shift in focus in the diagnosis of these infections from merely detecting presence/absence of infection towards detecting its impact on production. This has been facilitated by studies observing consistent negative correlations between helminth diagnostic test results and measures of productivity. Veterinarians are increasingly challenged to consider the economic aspects of their work, and the use of these tests should now be integrated in economic evaluation frameworks for improved decision making. In this paper, we review recent insights in the farm-specific economic impact of helminth infections on dairy cattle farms as well as in farmer attitudes and behaviour regarding helminth control. Combining better economic impact assessments of helminth infections together with a deeper understanding of the non-economic factors that drive a farmer’s animal health decisions should result in more effective control strategies and increased farmer satisfaction.

## Background

Cattle are parasitized by various helminth species, the most important being gastrointestinal nematodes (GIN), lungworms and liver fluke. These pathogens can cause severe disease, affect productivity in all classes of stock, and are amongst the most important production-limiting diseases of grazing ruminants. Essentially all herds/flocks in a grass-based production system are affected. Infections with GIN and liver fluke are more chronic and the major economic impact is due to sub-clinical infections causing reduced growth, milk yield and fertility [1]. Infections with lungworm are more acute and can place a sudden high economic burden on a farm due to mortalities and sharp decreases in milk yield [2].

Over the last decade, the pressure on farm income has further increased due to higher production costs and fluctuating output prices [3]. Subtle changes in production efficiency can make the difference between profit and loss. Efficient farming with an optimal management of inputs such as stock, feed, and labour has therefore become increasingly important. Animal health decisions have a significant impact on production efficiency, but are also subject to resource scarcity and budget constraints. Veterinarians are thus increasingly challenged to consider the economic aspects of their work for a farmer. Hence, economic evaluation frameworks are needed that can be integrated in decision making.

Once the economic value of specific animal health interventions can be demonstrated, veterinarians are faced with a second problem. How can they convince the farmer to implement their advice? Often the claim of an economic benefit will not be sufficient to induce a real change in farm management, even if it is grounded on solid scientific evidence. In other words, we need to understand the complete rationality of farmers’ behaviour in order to improve compliance with the provided advice [4].

In recent years, research in these 2 fields (i.e. economics and socio-psychology) is emerging in the field of animal health in general as well as in the field of helminth control in ruminants. In this paper, we discuss the diagnostic tools and methods that are available to assess the economic impact of helminth infections on (dairy) cattle farms as well as recent insights in farmer attitudes and behaviour that can help in the development of effective communication strategies to increase the uptake of proposed intervention strategies.

## Diagnosis to assess production impact

Over the last decade, an important paradigm shift occurred in the diagnosis of parasitic helminth infections in cattle. There has been a shift in focus from merely detecting presence/absence of infection towards detecting its impact on production. This is important because i) helminth infections are highly prevalent (“a cow without parasites is not a cow”) and ii) not every infection is of economic relevance. For example, fasciolosis is mostly a chronic disease, and often it is already known when the infection is around on the farm. Instead of demonstrating presence/absence of infection in a cow or herd, it is more relevant to identify the associated production losses to convince farmers that further diagnosis, and control measures, are worth considering [5]. This paradigm shift has been made possible by epidemiological research that observed consistent negative correlations between helminth diagnostic test results and measures of productivity.

In first-season grazing cattle, the serum pepsinogen concentration can be used to discriminate between different levels of *Ostertagia ostertagi*-infection and morbidity and associated production losses [6]. However, the lack of standardization between laboratories, the relatively high cost of the test and the fact that much of its informative value is lost soon after housing of the animals, when there is no new exposure to incoming infective larvae, are important constrains to its widespread uptake [7, 8]. Consequently, current research is investigating the value of *O. ostertagi* serum antibody levels in assessing production impacts, as it could overcome some of these drawbacks [9]. Faecal egg counts (FECs) of GIN correlate well with initial infection rates approximately 2 months after turnout on pasture for first-season grazing animals. However, after that period, host immunity reduces the correlation with actual worm burden and it seems impossible to indicate what weight gains are obtained by the end of the first grazing season, from FECs measured early in the season [10]. Therefore, FECs are primarily considered useful for understanding epidemiology rather than assessing infection levels or production impact [11].

In adult cattle, consistent negative relationships have been demonstrated between antibody levels to GIN or liver fluke in bulk tank milk and herd-average milk production [12–14]. In beef cattle, the quantification of antibody levels against GIN and liver fluke in meat juice obtained in the abattoir has been proposed, showing negative correlations with carcass weight and conformation score [15]. Studies have also shown negative relationships between helminth-specific antibody levels and reproduction and mortality indices at the herd level [16, 17]. Most recently, negative correlations have been established between a bulk tank milk ELISA for lungworm infection and milk production. Results showed a difference in milk production and milk fat of 1.0–1.7 kg/cow per day and 0.08–0.14 % between lungworm positive and negative herds, respectively [18], providing for the first time evidence of the economic importance of subclinical lungworm infections.

These established relationships can be used to indicate helminth-induced production losses associated with a test result of a specific farm. Several limitations with this approach remain, such as the lack of species-specificity of the measured antibody levels and the rather weak relationship between detected antibody levels and production responses after anthelmintic treatment. Nonetheless, it provides an ally to communicate to farmers on the importance (or not) of a helminth infection and to help monitor potential production losses [19].

## From production to economic impact

The impact of helminths on animal productivity is increasingly well understood [19], but the economic impact depends on multiple other factors such as farm-specific input and output prices and local regulations. The established links between diagnostics for different helminth infections with production losses, now allow to include this information in models that aim to assess the economic impact of the infection at regional or even farm-level. Such economic models of animal diseases are important because they contribute to balance expenditures on disease control with the actual disease costs and to evaluate the economic attractiveness of animal health interventions compared to other investment opportunities [20].

Considerable progress has been made in recent years with models to estimate the cost of helminth infections and/or interventions measures at the farm level. Some of these models are available to veterinarians at www.ParaCalc.com [21]. First, there is a deterministic spread-sheet model (“cost of worm infections”) where results from diagnostic methods (i.e. pepsinogen assay and serum ELISA for growing cattle and bulk tank milk ELISAs for adult cattle) to monitor the helminth infection status on a dairy herd and anthelmintic usage are used as input parameters. It produces a report with the expected annual loss due to infections with GIN and liver fluke to discuss with the farmer. The model is useful to evaluate the general importance of the infections, to monitor the evolution of costs across different years and to benchmark the results with peers. However, it does not consider the principle of “recoverable loss” [22]. How much of the total costs induced by helminth infections can be avoided by intervention measures? This is often difficult to determine because: (i) it is impossible to eliminate the infection from a farm; (ii) there can be remaining tissue damage after effective treatment or (iii) re-infection can occur at varying levels. Obtaining such information requires the set-up of experiments, by preference under commercial farming conditions, that evaluate the impact of specific intervention measures. Such information is increasingly available, especially to evaluate the production effects of strategic anthelmintic usage e.g. [23, 24], and this information was used to develop the second tool “treatment strategies against gastrointestinal worms” on ParaCalc.com. It estimates the likely economic benefit and uncertainty of a number of anthelmintic treatment strategies of adult cows and produces a report to discuss with the farmer.

Most recently, the farm-specific economic impact of helminth infections has also been studied using efficiency analysis [25]. Efficiency analysis studies the conversion of input(s) into output(s) and compares the current performance level of a farm with the performance level of peer farms with similar production technologies [26]. Using this approach, GIN infections appeared to mainly constrain the efficient transformation of pasture, health related costs and labour into milk. The inefficiency related with GIN infections was reduced when both high levels of concentrates, and also high levels of roughage were supplied [27]. Efficiency analysis has the potential to identify different improvement paths depending on the farm-specific production process and this was recently investigated by van der Voort et al. [28]. Farms were clustered in 3 groups depending on technical efficiency (TE) and input use. In low TE farms with a relatively low use of concentrates, there was no correlation between TE and level of exposure to GIN. Therefore, they are unlikely to improve economic performance by lowering the exposure to GIN infections. Analysis suggested they could best improve economic performance by making more use of concentrates. In farms with an intermediate TE and relatively high use of concentrates, there was a strong negative correlation between GIN exposure and TE. In addition, analysis showed that economic performance could be improved by substituting part of concentrates by grazing, which could lead to a higher infection pressure. This makes monitoring GIN infection and intervening by anthelmintic treatment when significant GIN exposure is observed, crucial in this group. In farms with the highest TE and intermediate use of concentrates, there was also a negative correlation between TE and GIN exposure. Analysis suggested that the economic performance could be improved by both reducing input use and reducing infection. In conclusion, efficiency analysis allows to establish links between animal disease and input use and input transformation. It can detect trade-offs and synergies between animal health interventions and input-output transformation. Whereas the implications of a vet’s advice are traditionally restrained to animal health issues and the improvement of technical key performance indicators, with this technique, we should be able to place our advice better in the whole-farm economic context. This approach is still in the research phase, but it is to be expected that it will be integrated in practical decision support tools for herd health management in the medium term.

## Non-economic factors that drive animal health decisions

Suppose that we have a high quality economic assessment report at hand to discuss with the farmer and that we are able to distil clear suggestions to improve his/her animal health management. Will this be sufficient for the farmer to implement our proposed strategies? It is now well understood that farmers’ decisions about their enterprises are not solely based on financial and business criteria. Farmers’ motives are rooted in deeply held values and also influenced by attitudes, beliefs and social norms [4]. Understanding all the values that drive farmer behaviour requires socio-psychological research, aimed to increase understanding of a farmer’s rationality and more effective advisory interventions [4, 29].

In the field of helminth control, Vande Velde et al. [30] investigated farmers’ intention to adopt diagnostic methods before implementing anthelmintic drugs in cattle. Based on two fundamental theories in the fields of behavioural and health psychology, a survey was carried out in 574 Flemish dairy farms to investigate the influence of the following variables: ‘attitude towards preventive use of anthelmintics’, ‘attitude towards diagnostic tools’, ‘subjective norms’, ‘behavioural control’ and ‘perceived risk’. The results showed that ‘attitude towards diagnostic methods’ and ‘subjective norms’; i.e. the influence of significant others, had the strongest, positive influence on adoption intention of diagnostic methods. ‘Attitude towards the preventive use of anthelmintic drugs’ had a negative effect on adoption intention of the diagnostic methods. ‘Perceived risk’, which was defined as the perceived susceptibility and severity of anthelmintic resistance on their farm, had no effect on the intention to adopt diagnostic methods. These results implicate that if we want to persuade farmers to make more use of diagnosis before anthelmintic treatment decisions are made, we should reinforce their positive attitude towards diagnosis and make use of their social network, which could implicate family, peer-farmers and the veterinarian. At present, the argument of anthelmintic resistance has no or little effect on dairy farmers’ intention to use diagnostics for helminth diseases, at least in this study population.

In order to investigate how veterinarians can improve their communication, we can learn from socio-psychological studies that have been conducted on different topics such as biosecurity, notification of notifiable diseases, antimicrobial usage and mastitis management [31–33]. Although the results cannot be extrapolated beyond their scope, similar patterns often emerge. Identification of different behavioural types is a first step towards better adapted advice and increased compliance. In the UK, Rehman et al. [34] differentiated farmers with a family orientation from entrepreneurs, life-stylers, hobbyists and independent farmers, respectively. In Brazil, Pereira et al. [35] were able to classify beef farmers that were considered receptive towards novel technology adoption based on their main sets of goals and values: the professional farmer, the committed environmentalist, the profit maximiser and the aspirant top farmer. The study showed a considerable diversity of values and goals even within this subset of progressive farmers. This diversity should be taken into account, because the advice that is in accordance with and reinforce the farmer’s core values will have the highest uptake. Age may also be an important criterion as Hamilton et al. [32] showed that young farmers (<45 years) tend be more entrepreneurial and amenable to change. Finally, the subjective norms, i.e. the social network surrounding the farmer, is often one of the most influential factors in driving animal health decisions [4, 36]. This can be exploited by the organization of farmer groups to provide a forum where farmers can explore management options and learn from each others’ views and experience [37]. Group learning is most successful if it includes experiential learning, group autonomy and builds on ongoing relationships and learning opportunities [38]. A catch can be that endemic livestock disease can be viewed as a problem for ‘bad’ farmers and not an issue for those individuals who manage their stock well. As such, there may be a low motivation to form groups to address what is largely perceived as an individual problem [39].

## Conclusion

Several diagnostic tools and methods are now available to assess the economic impact of helminth infections on (dairy) cattle farms. These include herd anamnesis in combination with serum pepsinogen assay and bulk tank milk ELISA for *O. ostertagi*, *F. hepatica* and *D. viviparus*. The use of these diagnostics is being integrated in decision support tools that should allow the veterinarian to estimate the economic consequences of his/her interventions and advice regarding helminth control. This can contribute in general to a ‘diagnosis before treatment’ approach and thus increase the sustainability of anthelmintic control by a better grounded and selective treatment [40].

Besides economic evaluations, more emphasis will have to be put on how advice is most effectively communicated. With the current plethora in websites, blogs and other communication channels, farmers are looking for trustworthy sources where they find reliable information that fits their situation. Private veterinarians are widely seen as such credible sources of information [36, 41]. Yet it appears that our communicative skills can still be improved. Farmers in general do not voluntarily communicate on their needs regarding animal health [42] and, therefore, veterinarians should actively seek those needs. Further, being more explicit during farm visits in discussing the farmer’s goals and priorities and providing a clear summary at the end of the visit of any advice given, would mean a significant step forward towards improved veterinary communication [42]. By understanding the core goals and values of different types of farmers, advice can be better targeted and framed in order to achieve higher compliance and farmer satisfaction.

## Abbreviations

FEC: Fecal egg count
GIN: Gastrointestinal nematodes
TE: Technical efficiency
